# A comprehensive global perspective on phylogenomics and evolutionary dynamics of *Small ruminant morbillivirus*

**DOI:** 10.1038/s41598-019-54714-w

**Published:** 2020-01-08

**Authors:** Muhammad Zubair Shabbir, Aziz-ul Rahman, Muhammad Munir

**Affiliations:** 1 University of Veterinary and Animal Sciences, Lahore, 54600 Pakistan; 2 Division of Biomedical and Life Sciences, Lancaster University, Lancaster, LA1 4YG United Kingdom

**Keywords:** Genetic databases, Viral evolution

## Abstract

A string of complete genome sequences of *Small ruminant morbillivirus* (SRMV) have been reported from different parts of the globe including Asia, Africa and the Middle East. Despite individual genome sequence-based analysis, there is a paucity of comparative genomic and evolutionary analysis to provide overarching and comprehensive evolutionary insights. Therefore, we first enriched the existing database of complete genome sequences of SRMVs with Pakistan-originated strains and then explored overall nucleotide diversity, genomic and residue characteristics, and deduced an evolutionary relationship among strains representing a diverse geographical region worldwide. The average number of pairwise nucleotide differences among the whole genomes was found to be 788.690 with a diversity in nucleotide sequences (0.04889 ± S.D. 0.00468) and haplotype variance (0.00001). The RNA-dependent-RNA polymerase (*L*) gene revealed phylogenetic relationship among SRMVs in a pattern similar to those of complete genome and the nucleoprotein (*N*) gene. Therefore, we propose another useful molecular marker that may be employed for future epidemiological investigations. Based on evolutionary analysis, the mean evolution rate for the complete genome, *N*, *P*, *M*, *F*, *H* and *L* genes of SRMV was estimated to be 9.953 × 10^–4^, 1.1 × 10^–3^, 1.23 × 10^–3^, 2.56 × 10^–3^, 2.01 × 10^–3^, 1.47 × 10^–3^ and 9.75 × 10^–4^ substitutions per site per year, respectively. A recombinant event was observed in a Pakistan-originated strain (KY967608) revealing Indian strains as major (98.1%, KR140086) and minor parents (99.8%, KT860064). Taken together, outcomes of the study augment our knowledge and current understanding towards ongoing phylogenomic and evolutionary dynamics for better comprehensions of SRMVs and effective disease control interventions.

## Introduction

Peste des petits ruminants (PPR), caused by *Small ruminant morbillivrus* (SRMV), is a contagious transboundary disease of domestic and wild ruminants^1,2^. Despite exhaustive vaccination, the disease is endemic across many regions/countries in Africa, Middle East and Asia, where occurrence of frequent disease outbreaks is not uncommon^3–7^. Currently, the PPR is threatening approximately 80% of the global population of sheep and goats with an estimated loss of USD 2.1 billion per year^8^.

The SRMV belongs to the genus *Morbillivirus* within the family *Paramyxoviridae*. It is a pleomorphic and enveloped virus that carries a negative sense RNA genome^9^ of variable length, from 15,927 to 16,058 nucleotides (NCBI database). The genome encodes six structural and two non-structural proteins in an order of 3′-N-P/C/V-M-F-HN-L-5′. Non-structural proteins (V and C) are encoded either by alternate open reading frames or mRNA editing in the phosphoprotein (P) gene. Based upon either *N* gene (255 bp) or *F* gene (322 bp), four distinct lineages of SRMVs (I-IV) are reported so far. Lineage I-II viruses are mostly reported from West African countries. Lineage III viruses seem restricted to the Middle East and East African countries. Lineage IV viruses have been reported from Asian and African countries^1,10^. The lineage IV is replacing prevalence of other lineages (i.e. I-III) territories and the occurrence of lineage IV is overwhelming even in Africa. These features demonstrate that lineage IV possess stronger positive selection and host-adaptation potential in a wide spectrum of hosts and geographical areas^11,12^.

Given the fact that genetic variations within a population of viruses could alter their pathogenicity and host spectrum, viral genetic diversity is considered a key to unleash viral evolution^13^. Using complete or partial sequencing of single genes (*H*, *N* or *F*), the clustering pattern, genomic and residue characteristics of SRMVs have widely been studied and discussed across the globe^5,10,12,14^. However, based upon each of these particular genes, the deduced genomic and residue characteristics may not be considered enough to predict ongoing evolutionary patterns across the whole length of the genome. In addition, many aspects of SRMVs evolution, including ancestral strain links, historical and geographic patterns of strain dispersal, divergence and time of origin remain poorly understood. These aspects are important because evolution within a single gene may not necessarily be occurring at the same rate as that of the whole genome^15^. Also, being RNA viruses, SRMVs are more prone to mutations during acute infection and therefore could present a polymorphic population^11^. Therefore, genetic diversity driven from consensus sequences of partial genomes could be far from representing the actual polymorphism across the whole length of the genome. Taken together, understanding comparative phylogenomics and evolutionary dynamics by exploiting complete genome sequence data of SRMVs facilitate better elucidate the genetic diversity, trends in its evolution and disease distribution pattern across diverse geographical regions. With this background, complimented by two complete genome sequences from Pakistan we used complete genome sequence data of SRMVs accessible in public database (until October 01, 2019) and analyzed for genetic diversity, phylogenomics and residue characteristics through different bioinformatics tools. We extend the analysis to each of the coding genes and identified potential ancestral relationship among SRMV-lineages reported from different countries during different time-period. In addition, we analyzed coding genes of all reported complete genomes to determine SRMV’s divergence time, and identified another candidate gene to be used as a phylogenetic marker. Together, the outcome will be expected to enhance our understating of phylogenetic and evolutionary dynamics of SRMVs across the globe.

## Results

### Comparative genome features

The comparative genomic analysis revealed a variable length of genomes as 15927, 15942, 15948, 15954, 15957 and 16058 nucleotides. Most of sequences across the globe had 15948 nucleotides (n = 39) whereas a number of Chinese strains (n = 31) and a Mongolian strain (KY888168) possessed a genome length of 15954 nucleotides. Only a single SRMV strain reported from India (KT270355) carried 15942 nucleotides. One Israeli strain (MF678816) had 15927 nucleotides. Two unusual genome lengths of 15957 (KM089831) and 16058 nucleotides (KM816619) were exclusively reported from China (Table 1). Excluding complete genomes of unusual lengths (MF678816, KM089831 and KM816619) while performing complete genome-specific analysis, the percentage for GC and AT contents was 47% and 53%, respectively. The proportion of GC content was found highest in *N* gene (50%) followed by *P* (48%), each of *M*, *F*, *H* (46%), *L* genes (43%), trailer (41%) and leader region (38%) (Table 2). The study genomes had a 52 nucleotide (nt) long leader in 3ʹ UTR at 107 nt long genome promoter region and a 73 nt long trailer at 5ʹ UTR in 109 nt long anti-genome promoter region. The total length of each of the genes varied across the whole genome: *N* gene (1578 nt) encoded 526 aa of 58 KDa, *P* gene (1530 nt) encoded 510 aa of 55 KDa, *M* gene (1008 nt) encoded 336 aa of 38 KDa, *F* gene (1641 nt) encoded 546 aa of 59 KDa, *H* gene (1830 nt) encoded 610 aa of 69 KDa and *L* gene (6552 nt) encoded 2184 aa of 247 KDa. Although all complete SRMV sequences showed variable genome length, the coding region for each of the genes was the same. The varying genome length was due to insertion of nucleotides in a non-coding region between *P* and *M* genes, and between *M* and *F* genes (Table 3). However, all genes were separated by similar conserved non-coding intergenic trinucleotide (CTT) except for one intergenic region between *L* gene and the trailer sequence (CTA).

**Table 1:** A brief summary of dataset on SRMVs available at public database incluidng under-study Pakistan-originated strains

Lineage	Geography	Accession number	Strain name	Genome length (nts)	Year	Host	Location
I	Africa	KP789375	E32/1969	15948	1969	Goat	Senegal
		EU267273	ICV89	15948	1989	Goat	Nigeria
II	Africa	MF741712	PPRV/Sierra Leone/048/2011	15948	2011	Goat	Sierra Leone
		KU236379	Lib/2015	15948	2015	Goat	Libya
		KR781451	CIV/01 P/2009	15948	2009	Goat	Cote d’Ivoire
		KM212177	SnDk11/13	15948	2013	Goat	Senegal
		KR781449	Benin/10/2011	15948	2011	Sheep	Benin
		KJ466104	Ghana/NK1/2010	15948	2010	Sheep	Ghana
		KR828814	NGKW2012–MSLN	15948	2012	Goat	Nigeria
		EU267274	Ng76/1	15948	1976	Goat	Nigeria
		KR781450	Benin/B1/1969	15948	1969	Goat	Benin
		HQ197753*	Nigeria/75/1	15948	1975	Goat	Nigeria
		X74443*	Nig/Vaccine	15948	1975	Goat	Nigeria
III	Asia	KJ867544	Oman 1983	15948	1983	Goat	Oman
		KJ867545	UAE 1986	15948	1986	Gazelle	UAE
	Africa	KJ867540	Ethiopia 1994	15948	1994	Goat	Ethiopia
		KJ867543	Uganda 2012	15948	2012	Goat	Uganda
IV	Africa	KR828813	NGYO2013–2162	15948	2013	Goat	Nigeria
		KJ867541	Ethiopia 2010	15948	2010	Goat	Ethiopia
		KY885100	S15	15948	2015	Goat	Algeria
		KC594074	Morocco 2008	15948	2008	Goat	Morocco
	Euro-asiatic	AJ849636	Turkey 2000	15948	2000	Goat	Turkey
	Asia	MF678816	1008	15927	2017	Nubian ibex	Israel
		MF737202	Georgia/Tbilisi/2016	15948	2016	Goat	Georgia
		KJ867542*	Sungri 1996 MSD	15948	1996	Goat	India
		KR140086	Izatnagar/94	15948	1994	Goat	India
		KF727981*	Sungri/96	15948	1996	Goat	India
		JX217850	Tibet/Bharal/2008	15948	2008	Bharal	China
		FJ905304	China/Tibet/Geg/07–30	15948	2007	Goat	China
		KX421388	China/33/2007	15948	2007	Goat	China
		JF939201	China/Tib/07	15948	2007	Goat	China
		KM816619	GZL-14	16058	2014	Goat	China
		KT633939	China/XJBZ/2015	15954	2015	Ibex	China
		KY888168	PPRV/Mongolia/9/2016	15954	2016	Goat	Mongolia
		KM089830	CH/HNNY/2014	15954	2014	Goat	China
		KM089832	CH/HNZM/2014	15954	2014	Goat	China
		KP868655	CH/GDDG/2014	15954	2014	Goat	China
		KM089831	CH/HNZK/2014	15957	2014	Goat	China
		KP260624	China/BJ/2014	15954	2014	Goat	China
		MF443343	ChinaJS2014	15954	2014	Goat	China
		MF443344	ChinaJL2014	15954	2014	Sheep	China
		MF443352	ChinaGD2014	15954	2014	Goat	China
		MF443346	ChinaHLJ2014	15954	2014	Goat	China
		MF443337	ChinaSX2014	15954	2014	Goat	China
		MF443353	ChinaCQ2014	15954	2014	Goat	China
		MF443339	ChinaSaX2014	15954	2014	Goat	China
		MF443345	ChinaHN2014	15954	2014	Goat	China
		MF443347	ChinaHeN2014	15954	2014	Goat	China
		MF443348	ChinaHB2014	15954	2014	Goat	China
		MF443336	ChinaYN2014	15954	2014	Goat	China
		MF443342	ChinaJX2014	15954	2014	Goat	China
		MF443335	ChinaZJ2014	15954	2014	Goat	China
		MF443338	ChinaSC2014	15954	2014	Goat	China
		MF443340	ChinaNX2014	15954	2014	Sheep	China
		MF443349	ChinaGZ2014	15954	2014	Goat	China
		MF443350	ChinaGX2014	15954	2014	Goat	China
IV	Asia	MF443354	ChinaAH2014	15954	2014	Goat	China
		KX421387	China/5/2013	15954	2013	Goat	China
		KX354359	PPRV-FY	15948	2015	Goat	China
		MF443341	ChinaLN2014	15954	2014	Goat	China
		MF443351	ChinaGS2014	15954	2014	Sheep	China
		KX421384	China/2/2013	15954	2013	Goat	China
		KX421386	China/4/2013	15954	2013	Goat	China
		KX421385	China/3/2013	15954	2013	Goat	China
		MG581412	PPRV/Bangladesh/BD2/2008	15948	2008	Goat	Bangladesh
		KM091959	China/XJYL/2013	15954	2013	Goat	China
		KT270355	IND/TN/GIN/2014/01	15942	2014	Goat	India
		KT860063	IND/TN/VM/2014/02	15948	2014	Goat	India
		KX033350	IND/Delhi/2016/05	15948	2016	Goat	India
		KT860064	IND/TN/VEL/2015/03	15948	2015	Sheep	India
		KR261605	India/TN/Gingee/2014	15948	2014	Goat	India
		KT860065	IND/TN/ED/2015/04	15948	2015	Sheep	India
		KY967608	SRMV/Lahore/UVAS/Pak/2015	15948	2015	Sheep	Pakistan
		KY967609	SRMV/Faisalabad/UVAS/Pak/2015	15948	2015	Goat	Pakistan
		KY967610	SRMV/Layyah/UVAS/Pak/2015	15948	2015	Goat	Pakistan

*Vaccine strains were excluded from any of the genomic and/or residue analysis performed in this manuscript.

**Table 2:** A brief descriptions on genome atlas including coding and non-coding regions of so far reported SRMVs worldwide

Genome regions	Position	Total length	GC%	3ʹUTR	ORF	5ʹUTR	Coding gene amino acid*	Intergenic trinucleotide region	Molecular weight (KDa)
Leader	1–52	52	38	—	—	—			
N	55–1744	1689	50	59	1578	52	526	CTT	58
P	1748–3402	1655	48	66	1530	59	510	CTT	55
M	3406–4888	1484	46	444	1008	32	336	CTT	38
F	4892–7306	2410	46	136	1641	633	546	CTT	59
H	7306–9262	1957	46	107	1830	20	610	CTT	69
L	9266–15908	6643	43	69	6552	22	2184	CTT	247
Trailer	15912–15948	37	41	—	—	—		CTA	

UTR: untranslated region, ORF: open reading frame *Including stop codon.

**Table 3:** A comparative analysis for the coding genes and intergenic regions present in the whole genome of SRMVs reported from different regions of the globe.

Regions	15942 nt	15948 nt	15954 nt	15957 nt	16058 nt
3ʹ UTR	107	107	107	107	107
N	108–1685	108–1685	108–1685	108–1685	108–1685
Non-coding (N-P)	123	123	123	123	123
P	1807–3336	1807–3336	1807–3336	1807–3336	1807–3336
Non-coding (P-M)	103	103	103	106	103
M	3438–4445	3438–4445	3438–4445	3441–4448	3438–4445
Non-coding (M-F)	1076	1082	1088	1088	1192
F	5520–7160	5526–7166	5532–7172	5535–7175	5636–7276
Non-coding (F-H)	161	161	161	161	161
H	7320–9149	7326–9155	7332–9161	7335–9164	7436–9265
Non-coding (H-L)	134	134	134	134	134
L	9282–15833	9288–15839	9294–15845	9297–15848	9398–15949
5ʹ UTR	109	109	109	109	109

### Percentage identity of nucleotide and comparative residue analysis

We found a varying nucleotide divergence among strains representing different lineages and geographical settings. For instance, a maximum nucleotide divergence (12.7%) was observed among Mongolian, Georgian (lineage IV) and Asian strains (lineage III). This was followed by 11.9% divergence between Pakistani (lineage IV) and other Asian strains (lineage III), and 11.8% divergence between Chinese (lineage IV) and rest of Asian strains (lineage III). As high as 11.5% nucleotide divergence was observed between Asian (lineage II) and African strains (lineage III) of SRMV. Similarly, a total of 11% nucleotide divergence was observed between African strains of lineages II and III. However, a variable divergence (8.5–10.3%) was noticed between SRMVs of lineage I and IV whereas, a divergence of 1.0–4.9% was revealed among strains within lineage IV (Table 4).

**Table 4:** Percntage nucleotide identities and divergence derived from complete genome consensus sequences of SRMVs strains (lineage I–IV) reported so far in the public database.

Lineages	SRMV strains	I	II		III		IV									
		Africa /1969–89	Africa /1969–76	Africa /2009–15	Asia /1983–86	Africa /1994–2012	India /1994–96	India /2014–16	China /2007–08	China /2013–15	Mongolia /2016	Bangladesh /2008	Pakistan /2015	Georgia /2016	Ethiopia /2010	Morocco /2008
I	Africa/1969–89		7.3	9.0	9.5	9.5	8.5	9.4	9.2	9.6	9.9	10.3	9.5	9.7	9.4	9.2
II	Africa/1969–76	92.7		4.1	10.0	9.7	6.5	7.5	7.2	7.4	7.9	8.3	7.6	7.8	7.4	7.3
	Africa/2009–15	91	95.9		11.2	11.0	8.2	9.0	8.8	8.9	9.4	9.9	9.1	9.3	8.9	8.9
III	Asia/1983–86	90.5	90	88.8		3.1	11.0	11.7	11.4	11.8	12.1	12.6	11.9	12.1	11.7	11.5
	Africa/1994–2012	90.5	90.3	88.0	94.9		10.8	11.6	11.4	11.7	11.9	12.6	11.7	11.8	11.5	11.4
IV	India/1994–96	91.5	93.5	91.8	88.0	89.2		2.4	1.9	2.9	2.9	3.5	2.4	2.9	2.4	2.2
	India/2014–16	90.6	92.5	90.0	88.3	88.4	97.6		1.8	3.6	3.5	3.4	2.9	4.1	3.7	3.5
	China/2007–08	90.8	92.8	91.2	88.6	88.6	98.1	98.2		3.0	2.9	2.9	2.7	3.7	3.2	3.0
	China/2013–15	90.4	92.6	91.1	88.2	88.3	97.1	96.4	97		1.0	4.7	3.6	4.6	4.2	3.9
	Mongolia/2016	90.1	92.9	90.6	87.9	88.1	97.1	96.5	97.1	99		4.6	3.5	4.6	4.1	3.9
	Bangladesh/2008	89.7	91.7	90.1	87.4	87.4	96.5	96.6	97.1	95.3	95.4		4.1	5.2	4.8	4.6
	Pakistan/2015	90.5	92.4	90.9	88.1	88.3	97.6	97.1	97.3	96.4	97.5	95.9		4.2	3.6	3.5
	Georgia/2016	90.3	92.2	90.7	87.9	88.2	97.1	95.9	96.3	95.4	95.4	94.8	95.8		2.0	3.0
	Ethiopia/2010	90.6	92.6	91.1	88.3	88.5	97.6	96.3	96.8	95.8	95.9	95.2	96.4	98		2.0
	Morocco/2008	90.8	92.7	91.1	88.5	88.6	97.8	96.5	97	96.1	96.1	95.4	96.5	97	98	

**Figure 1:**
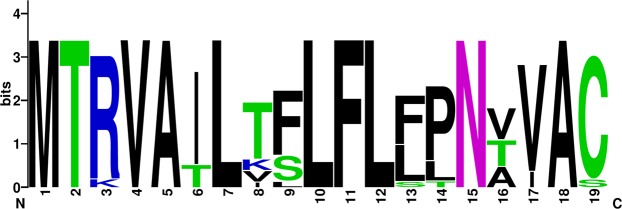
WebLogo-based diversity and/or conserveness of residues in proposed stretch at fusion protein of so-far reported SRMVs

Comparative residue analysis of different proteins across the entire genome length revealed conserved functional and/or structural motifs; however, few substitutions were noticed in some of the studied strains. A hypervariable region of varying length was observed in each of the SRMV proteins i.e., 423–456 aa in N, 74–111 aa in P, 73–197 aa in M, 6–16 aa in F, 174–179 aa in H and 617–627 aa in L protein. The nuclear export and nuclear localization signal, and RNA binding motifs appeared conserve in N protein of all strains. In P protein, a Soyuz 1 motif was also conserved in all strains except for the consensus sequence of Africa/1994–2012 (lineage III) where a total of six substitutions (L5Q, V10N, E11K, A14E, L16I and F20K) were observed. A serine residue (^151^S) in the P protein and a cell membrane anchor in the M protein were conserved in all of the SRMV sequences (Table 5). The signal peptide in F protein has previously been reported to be hypervariable (Table 6); however, while comparing SRMVs of different lineages, we proposed a relatively conserved long stretch of residue (^1^MTRVAILTFLFLFPNVVAC^19^) (Fig. 1). The cleavage motif (^103^GRRTRR^108^) was conserved in the F protein of all sequences. The fusion peptide motif was conserved for 109–133 aa in all SRMV strains except for consensus sequence of China/2013–15 strain where, phenylalanine (F) was replaced by leucine (L) at 1^st^ position of the motif. Substitutions were observed in leucin zipper domain of consensus sequence in lineage II (African/2009–15, V479I), lineage III (African/1994–2012, I463V) and lineage IV (Bangladesh/2008, A464T). All consensus strains from lineage II including China/2013–15, Mongolia/2016, Georgia/2016 and Ethiopia/2010 carried a conserved residue pattern for hydrophobic anchor membrane of F protein; however, two substitutions (A486V and G489S) were observed predominantly in sequences from lineage IV. While comparing residue type and position in the H protein, several substitutions were revealed. For strains within lineage IV, these included a substitution in the N-terminal anchor of an Indian strain (India/2014–16, A41V) and in Georgian strain (Georgia/2016, Y481H). A substitution common to all SRMV strains within lineage III was observed in SLAM binding site where tyrosine (Y) was replaced by phenylalanine (F) at position 553, whereas a substitution in asparagine N-linked glycosylation site (^215^NVT^217^) was exclusive to strains reported from Africa during 1994–2012 (Table 7). For the N protein, all functionally and structurally important motifs were conserved in strains representing lineage I-IV.

**Table 5:** A summarized comparative residue analysis of important domain and motif at NP, P and M proteins of SRMVs for their structural, functional and biologic activities.

Lineage	SRMV strains	Nucleocapsid protein			Phosphoprotein		Matrix protein
		NES (^4^LLKSLALF^11^)	NLS (^70^TGVMISML^77^)	RNA binding motif (^324^FSAGAYPLLWSYAMG^338^)	Soyuz 1 motif (^4^EQAYHVNKGLECIKSLK^20^)	Serine ^151^S	Cell membrane anchor (^50^FMYL^53^)
I	Africa/1969–89	^4^……..^11^	^70^……..^77^	^324^……………^338^	^4^……………..^20^	—	^50^….^53^
II	Africa/1969–76	^4^……..^11^	^70^……..^77^	^324^……………^338^	^4^……………..^20^	—	^50^….^53^
	Africa/2009–15	^4^……..^11^	^70^……..^77^	^324^……………^338^	^4^……………..^20^	—	^50^….^53^
III	Asia/1983–86	^4^……..^11^	^70^……..^77^	^324^……………^338^	^4^……………..^20^	—	^50^….^53^
	Africa/1994–2012	^4^……..^11^	^70^……..^77^	^324^……………^338^	^4^.L….VE..A.L...F^20^	—	^50^….^53^
IV	India/1994–96	^4^……..^11^	^70^……..^77^	^324^……………^338^	^4^……………..^20^	—	^50^….^53^
	India/2014–16	^4^……..^11^	^70^……..^77^	^324^……………^338^	^4^……………..^20^	—	^50^….^53^
	China/2007–08	^4^……..^11^	^70^……..^77^	^324^……………^338^	^4^……………..^20^	—	^50^….^53^
	China/2013–15	^4^……..^11^	^70^……..^77^	^324^……………^338^	^4^……………..^20^	—	^50^….^53^
	Mongolia/2016	^4^……..^11^	^70^……..^77^	^324^……………^338^	^4^……………..^20^	—	^50^….^53^
	Bangladesh/2008	^4^……..^11^	^70^……..^77^	^324^……………^338^	^4^……………..^20^	—	^50^….^53^
	Pakistan/2015	^4^……..^11^	^70^……..^77^	^324^……………^338^	^4^……………..^20^	—	^50^….^53^
	Israel/2017	^4^……..^11^	^70^……..^77^	^324^……………^338^	^4^……………..^20^	—	^50^….^53^
	Georgia/2016	^4^……..^11^	^70^……..^77^	^324^……………^338^	^4^……………..^20^	—	^50^….^53^
	Ethiopia/2010	^4^……..^11^	^70^……..^77^	^324^……………^338^	^4^……………..^20^	—	^50^….^53^
	Morocco/2008	^4^……..^11^	^70^……..^77^	^324^……………^338^	^4^……………..^20^	—	^50^….^53^

Note: Consensus sequences used in different lineages according to complete genome of strains; Asia/1983–86 in lineage III is consensus sequence of two strains including UAE/1986 (KJ867545) and Oman/1983 (KJ867544). Identical residues are shown as “.”.

**Table 6:** A summarized comparative residue analysis of important domain and motif in the F and H proteins of SRMVs for their structural, functional and biologic activities.

Lineage	SRMV strains	Fusion protein					Haemagglutinin protein			
		Signal peptide (^1^MTRVAILAFLFLFLNAVAC^19^)	Cleavage site (^103^GRRTRR^108^)	Fusion peptide (^109^FAGAVLAGVALGVATAAQITAGVAL^133^)	Leucin zipper domain (^459^LGNAVTRLENAKELLDASDQIL^480^)	Hydrophobic anchor membrane (^485^GVPFSGNMYIALAACIGVSLGLVTLICCKGRC^517^)	N terminal anchor (^35^PYILLGVLLVMFLSLIGLLAIAG^58^)	Histidine ^481^H	SLAM binding sites (^529^Y, ^530^D, ^533^R, ^552^F, ^553^Y, ^554^P)	Asparagine N–linked glycosylation (^215^NVS^217^, ^279^NMS^281^, ^395^NGT^397^)
I	Africa/1969–89	^1^….T.T..…P…..^19^	^103^.……^108^	^109^……………………^133^	^459^………………….^480^	^485^….G..L..G……………….….^517^	^35^…………………..^58^	—	^529^., ^530^., ^533^., ^552^., ^553^., ^554^.	^215^…^217^, ^279^…^281^, ^395^…^397^
II	Africa/1969–76	^1^…..T.V..…P.T…^19^	^103^.……^108^	^109^……………………^133^	^459^………………….^480^	^485^…………………….………^517^	^35^…………………..^58^	—	^529^., ^530^., ^533^., ^552^., ^553^., ^554^.	^215^…^217^, ^279^…^281^, ^395^…^397^
	Africa/2009–15	^1^…..T.VL.…PNT…^19^	^103^.……^108^	^109^……………………^133^	^459^………………V.^480^	^485^……………………………^517^	^35^…………………..^58^	—	^529^., ^530^., ^533^., ^552^., ^553^., ^554^.	^215^…^217^, ^279^…^281^, ^395^…^397^
III	Asia/1983–86	^1^…….TS…LT.T..S^19^	^103^.……^108^	^109^……………………^133^	^459^………………….^480^	^485^…L…L..G…………………..^517^	^35^…………………..^58^	—	^529^., ^530^., ^533^., ^552^., ^553^F, ^554^.	^215^…^217^, ^279^…^281^, ^395^…^397^
	Africa/1994–2012	^1^..K….TS…LPNT…^19^	^103^.……^108^	^109^……………………^133^	^459^….I……………..^480^	^485^…….L..G………………R…^517^	^35^…………………..^58^	—	^529^., ^530^., ^533^., ^552^., ^553^F, ^554^.	^215^..T^217^, ^279^…^281^, ^395^…^397^
IV	India/1994–96	^1^…….T..…P…..19	^103^.……^108^	^109^……………………^133^	^459^………………….^480^	^485^….G……………………….^517^	^35^…………………..^58^	—	^529^., ^530^., ^533^., ^552^., ^553^., ^554^.	^215^…^217^, ^279^…^281^, ^395^…^397^
	India/2014–16	^1^…….TS…LP.V…^19^	^103^.……^108^	^109^……………………^133^	^459^………………….^480^	^485^.A..G............................^517^	^35^……A…………….^58^	—	^529^., ^530^., ^533^., ^552^., ^553^., ^554^.	^215^…^217^, ^279^…^281^, ^395^…^397^
	China/2007–08	^1^…….T.…LP.V…^19^	^103^.……^108^	^109^……………………^133^	^459^………………….^480^	^485^.A..G………………………^517^	^35^…………………..^58^	—	^529^., ^530^., ^533^., ^552^., ^553^., ^554^.	^215^…^217^, ^279^…^281^, ^395^…^397^
	China/2013–15	^1^…….T..…P.V…^19^	^103^.……^108^	^109^L…………………^133^	^459^………………….^480^	^485^…………………….………^517^	^35^…………………..^58^	—	^529^., ^530^., ^533^., ^552^., ^553^., ^554^.	^215^…^217^, ^279^…^281^, ^395^…^397^
	Mongolia/2016	^1^…….T..…P.V…^19^	^103^.……^108^	^109^……………………^133^	^459^………………….^480^	^485^…………………….………^517^	^35^…………………..^58^	—	^529^., ^530^., ^533^., ^552^., ^553^., ^554^.	^215^…^217^, ^279^…^281^, ^395^…^397^
	Bangladesh/2008	^1^…….I.…LP.V…^19^	^103^.……^108^	^109^……………………^133^	^459…..^A…………….^480^	^485^.A..G……………………….^517^	^35^…………………..^58^	—	^529^., ^530^., ^533^., ^552^., ^553^., ^554^.	^215^…^217^, ^279^…^281^, ^395^…^397^
	Pakistan/2015	^1^…….TS.…P.V…^19^	^103^.……^108^	^109^……………………^133^	^459^………………….^480^	^485^.A..G……………………….^517^	^35^…………………..^58^	—	^529^., ^530^., ^533^., ^552^., ^553^., ^554^.	^215^…^217^, ^279^…^281^, ^395^…^397^
	Israel/2017	^1^…….T..…P.....^19^	^103^.……^108^	^109^……………………^133^	^459^………………….^480^	^485^….G………….F……….R…^517^	^35^…………………..^58^	—	^529^., ^530^., ^533^., ^552^., ^553^., ^554^.	^215^…^217^, ^279^…^281^, ^395^…^397^
	Georgia/2016	^1^…….K………..^19^	^103^.……^108^	^109^……………………^133^	^459^………………….^480^	^485^…………………….………^517^	^35^…………………..^58^	^481^Y	^529^., ^530^., ^533^., ^552^., ^553^., ^554^.	^215^…^217^, ^279^…^281^, ^395^…^397^
	Ethiopia/2010	^1^…….K……….^19^	^103^.……^108^	^109^……………………^133^	^459^………………….^480^	^485^…………………….………^517^	^35^…………………..^58^	—	^529^., ^530^., ^533^., ^552^., ^553^., ^554^.	^215^…^217^, ^279^…^281^, ^395^…^397^
	Morocco/2008	^1^…….T….S…I..^19^	^103^.……^108^	^109^……………………^133^	^459^………………….^480^	^485^….G……………………….^517^	^35^…………………..^58^	—	^529^., ^530^., ^533^., ^552^., ^553^., ^554^.	^215^…^217^, ^279^…^281^, ^395^…^397^

Note: Consensus sequences used in different lineages according to complete genome of strains; Asia/1983–86 in lineage III is consensus sequence of two strains including UAE/1986 (KJ867545) and Oman/1983 (KJ867544). Identical residues are shown as “.”.

**Table 7:** A summarized comparative residue analysis of important domain and motif in the L protein of SRMVs for their structural, functional and biologic activities.

Lineage	SRMV strains	Domain interact with P protein (^9^VLYPEVHLDSPIV^21^)	RNA binding motif (^540^KETGRLFAKMTYKM^553^)	Domain I (^659^FITADLKKYCLNWRYCL^679^)	Domain II (^731^FIKYPMGGIEGYCQKLWTISTIPYL^755^)	Domain III (^768^SLVQGDNQTIAVTK^781^)	Domain IV (^838^YDGLLVSQSLKSIAR^852^)	Polymerase associated motif		ATP binding site (^1766^K_21_GEGSGSM^1794^)	Methyltransferase associated motifs (^1766^K, ^1881^D, ^1917^K, ^1788^GEGSGSM^1974^, ^1809^YNSG^1812^, ^1855^TWVG^1858^)
	Africa/1969–89	^9^………….^21^	^540^…………..^553^	^659^……………..^679^	^731^…………………….^755^	^768^…………..^781^	^838^……………^852^	^771^QGDNQ^775^	^1464^GDDD^1467^	^1766^……..^1794^	^1766^., ^1881^., ^1917^., ^1788^…….^1974^, ^1809^….^1812^, ^1855^….^1858^
I	Africa/1969–76	^9^………….^21^	^540^…………..^553^	^659^……………..^679^	^731^…………………….^755^	^768^…………..^781^	^838^……………^852^	^771^…..^775^	^1464^….^1467^	^1766^……..^1794^	^1766^., ^1881^., ^1917^., ^1788^…….^1974^, ^1809^….^1812^, ^1855^….^1858^
II	Africa/2009–15	^9^………….^21^	^540^…………..^553^	^659^……………..^679^	^731^…………………….^755^	^768^…………..^781^	^838^……………^852^	^771^…..^775^	^1464^….^1467^	^1766^……..^1794^	^1766^., ^1881^., ^1917^., ^1788^…….^1974^, ^1809^….^1812^, ^1855^….^1858^
	Asia/1983–86	^9^………….^21^	^540^…………..^553^	^659^……………..^679^	^731^…………………….^755^	^768^…………..^781^	^838^……………^852^	^771^…..^775^	^1464^….^1467^	^1766^……..^1794^	^1766^., ^1881^., ^1917^., ^1788^…….^1974^, ^1809^….^1812^, ^1855^….^1858^
III	Africa/1994–2012	^9^………….^21^	^540^…………..^553^	^659^……………..^679^	^731^…………………….^755^	^768^…………..^781^	^838^……………^852^	^771^…..^775^	^1464^….^1467^	^1766^……..^1794^	^1766^., ^1881^., ^1917^., ^1788^…….^1974^, ^1809^….^1812^, ^1855^….^1858^
	India/1994–96	^9^………….^21^	^540^…………..^553^	^659^……………..^679^	^731^…………………….^755^	^768^…………..^781^	^838^……………^852^	^771^…..^775^	^1464^….^1467^	^1766^……..^1794^	^1766^., ^1881^., ^1917^., ^1788^…….^1974^, ^1809^….^1812^, ^1855^….^1858^
IV	India/2014–16	^9^………….^21^	^540^…………..^553^	^659^……………..^679^	^731^…………………….^755^	^768^…………..^781^	^838^……………^852^	^771^…..^775^	^1464^….^1467^	^1766^……..^1794^	^1766^., ^1881^., ^1917^., ^1788^…….^1974^, ^1809^….^1812^, ^1855^….^1858^
	China/2007–08	^9^………….^21^	^540^…………..^553^	^659^……………..^679^	^731^…………………….^755^	^768^…………..^781^	^838^……………^852^	^771^…..^775^	^1464^….^1467^	^1766^……..^1794^	^1766^., ^1881^., ^1917^., ^1788^…….^1974^, ^1809^….^1812^, ^1855^….^1858^
	China/2013–15	^9^………….^21^	^540^…………..^553^	^659^……………..^679^	^731^…………………….^755^	^768^…………..^781^	^838^……………^852^	^771^…..^775^	^1464^….^1467^	^1766^……..^1794^	^1766^., ^1881^., ^1917^., ^1788^…….^1974^, ^1809^….^1812^, ^1855^….^1858^
	Mongolia/2016	^9^………….^21^	^540^…………..^553^	^659^……………..^679^	^731^…………………….^755^	^768^…………..^781^	^838^……………^852^	^771^…..^775^	^1464^….^1467^	^1766^……..^1794^	^1766^., ^1881^., ^1917^., ^1788^…….^1974^, ^1809^….^1812^, ^1855^….^1858^
	Bangladesh/2008	^9^………….^21^	^540^…………..^553^	^659^……………..^679^	^731^…………………….^755^	^768^…………..^781^	^838^……………^852^	^771^…..^775^	^1464^….^1467^	^1766^……..^1794^	^1766^., ^1881^., ^1917^., ^1788^…….^1974^, ^1809^….^1812^, ^1855^….^1858^
	Pakistan/2015	^9^………….^21^	^540^…………..^553^	^659^……………..^679^	^731^…………………….^755^	^768^…………..^781^	^838^……………^852^	^771^…..^775^	^1464^….^1467^	^1766^……..^1794^	^1766^., ^1881^., ^1917^., ^1788^…….^1974^, ^1809^….^1812^, ^1855^….^1858^
	Israel/2017	^9^………….^21^	^540^…………..^553^	^659^……………..^679^	^731^…………………….^755^	^768^…………..^781^	^838^…..I………^852^	^771^…..^775^	^1464^….^1467^	^1766^……..^1794^	^1766^., ^1881^., ^1917^., ^1788^…….^1974^, ^1809^….^1812^, ^1855^….^1858^
	Georgia/2016	^9^………….^21^	^540^…………..^553^	^659^……………..^679^	^731^…………………….^755^	^768^…………..^781^	^838^……………^852^	^771^…..^775^	^1464^….^1467^	^1766^……..^1794^	^1766^., ^1881^., ^1917^., ^1788^…….^1974^, ^1809^….^1812^, ^1855^….^1858^
	Ethiopia/2010	^9^………….^21^	^540^…………..^553^	^659^……………..^679^	^731^…………………….^755^	^768^…………..^781^	^838^……………^852^	^771^…..^775^	^1464^….^1467^	^1766^……..^1794^	^1766^., ^1881^., ^1917^., ^1788^…….^1974^, ^1809^….^1812^, ^1855^….^1858^
	Morocco/2008	^9^………….^21^	^540^…………..^553^	^659^……………..^679^	^731^…………………….^755^	^768^…………..^781^	^838^……………^852^	^771^…..^775^	^1464^….^1467^	^1766^……..^1794^	^1766^., ^1881^., ^1917^., ^1788^…….^1974^, ^1809^….^1812^, ^1855^….^1858^
	Africa/1969–89	^9^………….^21^	^540^…………..^553^	^659^……………..^679^	^731^…………………….^755^	^768^…………..^781^	^838^……………^852^	^771^…..^775^	^1464^….^1467^	^1766^……..^1794^	^1766^., ^1881^., ^1917^., ^1788^…….^1974^, ^1809^….^1812^, ^1855^….^1858^

Note: Consensus sequences used in different lineages according to complete genome of strains; Asia/1983–86 in lineage III is consensus sequence of two strains including UAE/1986 (KJ867545) and Oman/1983 (KJ867544). Identical residues are shown as “.”.

### Estimation of evolutionary and divergence rates

Using a Bayesian coalescent approach, a molecular clock analysis of the whole genome and all coding gene sequences was performed to estimate the mean rate of evolution. Based on this analysis, the mean evolution rate for the complete genome of SRMV was estimated to be 9.953 × 10^–4^ substitutions per site per year. Best growth model was used for individual SRMV gene dataset to estimate the TMRCA and substitution rate per site per year. A cumulative interpretation of individual gene-based analysis showed rates of evolution in *N*, *P*, *M*, *F*, *H* and *L* genes as 1.1 × 10^–3^, 1.23 × 10^–3^, 2.56 × 10^–3^, 2.01 × 10^–3^, 1.47 × 10^–3^ and 9.75 × 10^–4^ site per year, respectively. The *N* gene (1.1 × 10^–3^) showed a lesser evolution rate as compared to other genes whereas it was highest for the *L* gene (9.75 × 10^–4^).

### Phylogenetic topology based on geographical pattern

**Figure 2:**
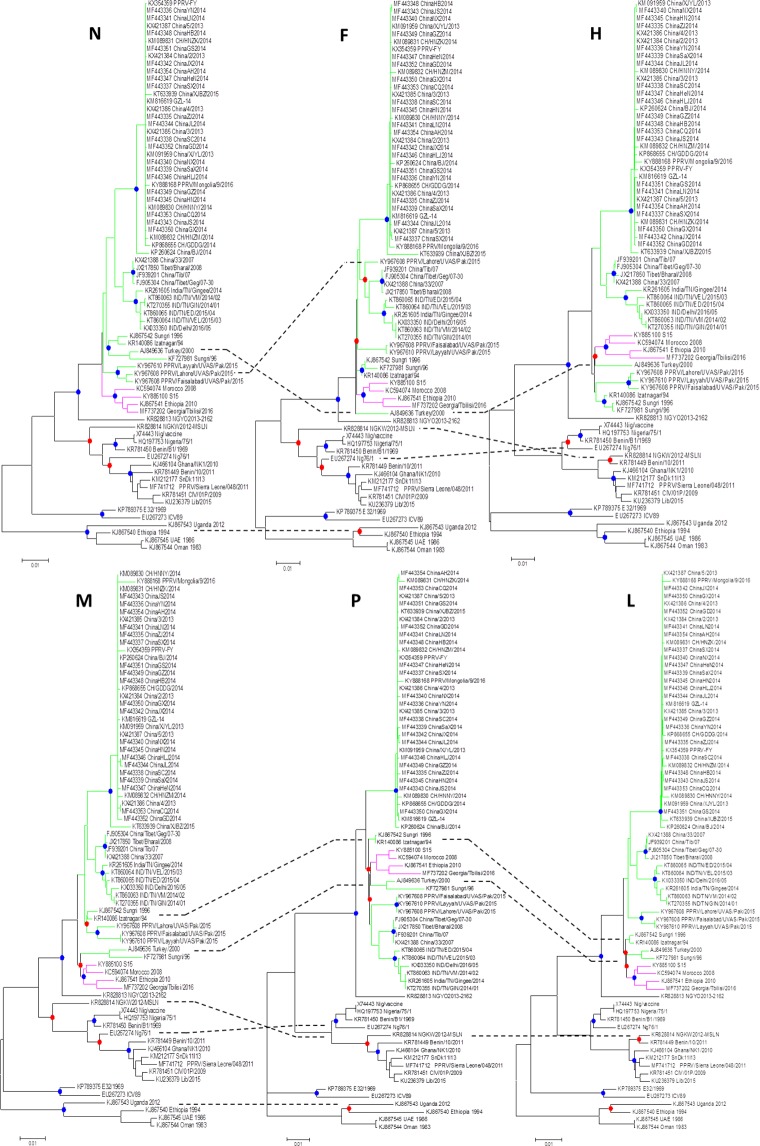
Individual coding gene-based phylogenetic analysis of so-far reported SRMVs revealed mismatching for monophyletic clustering of strains.

Utilizing each of the coding genes, the phylogenetic analysis of SRMV sequences revealed a distinct pattern of clustering according to the geographical locations. However, the complete *N* gene*-*based clustering pattern was more authoritative and conclusive followed by *L*, *H*, *F*, *M* and *P* genes (Fig. 2a,b). Within lineage II viruses, variations in clustering pattern were related to the reporting period from different regions in the African continent. In contrast, lineage III viruses from Africa showed variations in their clustering pattern on the basis of each of five gene used for analysis. For lineages IV viruses, there were significant variations in clustering pattern for each of the coding gene. The clustering pattern derived from *N* and *L* genes was similar to *M*, *P*, *F* and *H* genes. Since *N* and *L* genes-based topology of phylogenetic relationship among geographically distinct strain was found to be more precise and conclusive, the* L* gene is suggested to be employed in future epidemiological investigations.

**Figure 3:**
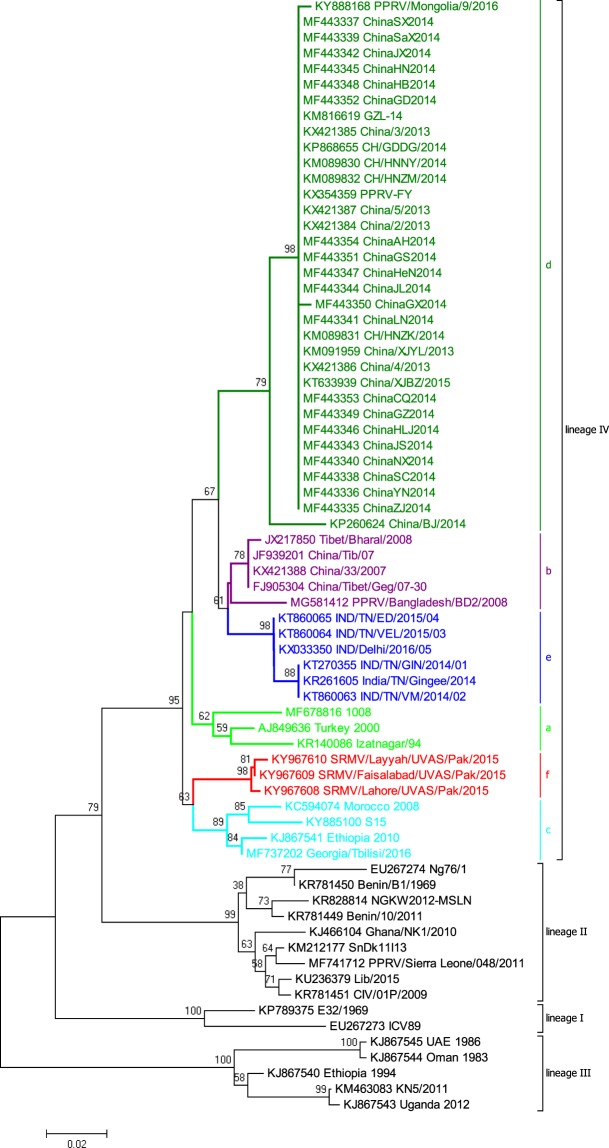
The complete *N* gene-based intra-lineage classification of strains within lineage IV.

Based on the analysis of the complete *N* gene dataset, we proposed a geography and timeline based-classification of SRMV strains within lineage IV. A substantial analysis revealed a 6% and 2% nucleotide divegernce as a considerable cut-off criterion for the clasification of SRMV lineages and sub-lineages, respectively. Further analysis identified a total of six sub-clades (a-f) where sub-clade “a” represented strains from India, Turkey and Israel during 1994–2017, sub-clade “b” contained Chinese strains reported in 2007–08, sub-clade “c” represented strains from Africa and Georgia during 2008–2016, sub-clade “d” had Chinese strains reported during 2013–2016, sub-clade “e” possessed strains reported from India during 2014–2016, and sub-clade “f” was exclusive to Pakistan-originated strains which were reported in 2015 (Fig. 3).

### Nucleotide diversity and selective pressure analysis

**Figure 4:**
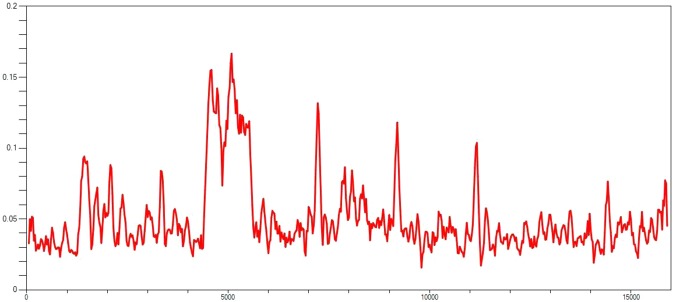
Nucleotide diversity plotamong whole genome sequences of SRMVs derived from DnaSP.

The average nucleotide diversity (Pi-value) was 0.04889 for complete genome of all SRMV strains. With a variance (0.00001) and standard deviation (0.002) for haplotype diversity (Hd = 1.000), the average nucleotide differences among all haplotypes was found to be k = 788.690. A total of 5891 mutations were observed in DnaSP analysis, where 10831 were monomorphic and 5117 were polymorphic. The polymorphic mutations consisted of 1311 singleton variable sites with 3806 parsimony informative sites. While an assessment for neutrality, the Tajima’s D value was found to be negative for all genes (*p* > 0.10). The reliability of the analysis, as determined by HKA test, was found to be 6.078 (X-square value) in T = 6.732 (divergence time) at a significant level (*p* = 0.0131) (Table 8). An analysis of the genetic diversity within the coding genes across the whole length of the genome revealed an occurrence of hotspot event (300 nt window size per ten nt overlapping steps) between 5ʹ UTR of *M* gene and 3ʹ UTR of *F* gene (Fig. 4). The nucleotide diversity across the coding genes of nucleotide sequence haplotypes was found to be highest in *H* gene (0.05171) followed by *P* (0.04527), *F* (0.04409), *N* (0.04068), *L* (0.03982) and *M* (0.03931) genes. On the other hands, the haplotype diversity (Hd) was observed to be higher in *L* gene (0.996) followed by *F* (0.926), *P* (0.921), *H* (0.920), *N* (0.909) and *M* (0.900) genes (Table 8).

**Table 8:** A brief description on genome polymorphism for secletion sites in the complete genome and each of the coding regions in SRMVs.

Parameters	Complete genome	N	P	M	F	H	L
Numbers of sites	15948	1578	1530	1008	1641	1830	6552
Monomorphic sites	10831	1115	1031	738	1168	1199	4754
Polymorphic sites	5117	463	499	270	473	631	1798
Total no. of mutation	5891	496	532	288	497	649	1893
Singleton variable sites	1311	115	171	71	118	161	477
Parsimony Informative Sites	3806	348	328	199	355	470	484
No. of haplotypes (h)	68	51	50	47	49	50	67
Haplotype diversity (Hd)	1.000	0.909	0.921	0.900	0.926	0.920	0.996
Variance of haplotype diversity	0.00001	0.00096	0.00054	0.00093	0.00076	0.00036	0.00001
Standard deviation of gene diversity	0.002	0.030	0.023	0.030	0.028	0.019	0.003
Nucleotide diversity (Pi)	0.04889	0.04068	0.04527	0.03931	0.04409	0.05171	0.03982
Standard deviation of Pi	0.00468	0.00465	0.00497	0.00442	0.00500	0.00568	0.00454
Average no. of pairwise nucleotide difference (k)	788.690	64.358	69.279	39.705	72.406	94.751	261.289
Tajima’ D	−1.23601	−1.31743	−1.38728	−1.24841	−1.10701	−0.85748	−1.21260

Note: HKA test direct mode: Divergence time T = 6.732 × –square value = 6.078, *P* value = 0.0131*, * = 0.01 < *p* < 0.05.

**Figure 5:**
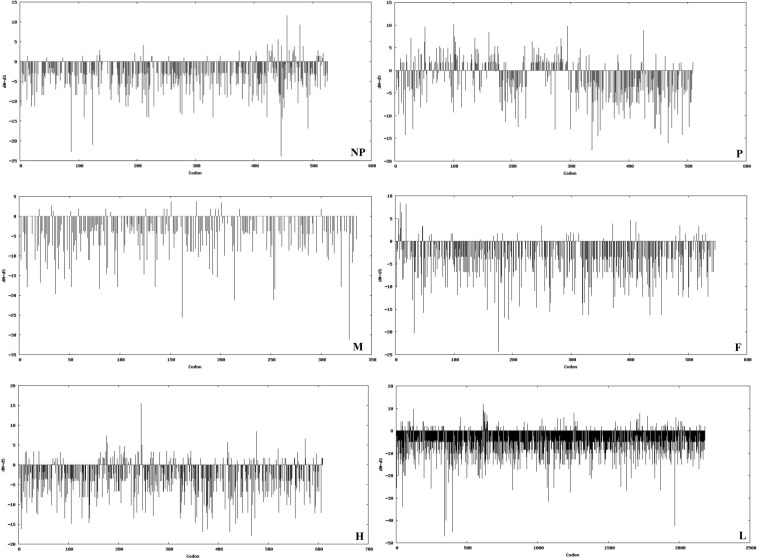
Differences in codon position, synonymous and non-synonymous substitutions (dN-dS values) for each of individual genes.

Datamonkey output for selective pressure analysis across CDS regions is summarized in Table 9. Although none of the gene carried a mean dN-dS greater than 1 at *p* < 0.05, it was highest for *P* gene (0.44679) followed by *H* (0.20017), *N* (0.12168), *F* (0.10253), *L* (0.08976) and *M* (0.06601) genes. At *p* < 0.05, analyzing through different algorithmic approaches (SLAC, FEL, IFEL, REL and MEME), revealed that the *L* gene showed a highest positive selection sites (96) followed by *N* (27), *F* (21), *P* (16), *H* (12) and *M* (2) genes. The plots against codon positions for individual genes were drawn using SLAC statistical approach based on dN-dS value (Fig. 5).

**Table 9:** Data Monkey analysis based brief summary of positive and negative substitution sites in each of the coding gene of so far reported SRMVs.

Parameters	N	P	M	F	H	L
Mean dN–dS	0.12168	0.44679	0.06601	0.10253	0.20017	0.08976
No. of duplicate sequences	18	22	24	22	19	5
**Single Likelihood Ancestor Counting (SLAC)**						
No. of positive and negative selection sites along with codon position at 95% confidence level	1 positive (456) and 48 negative sites	0 positive and 13 negative sites	0 positive and 29 negative sites	0 positive and 48 negative sites	1 positive (246) and 41 negative sites	0 positive and 149 negative sites
**Fixed Effect Likelihood (FEL)**						
No. of positive and negative selection sites along with codon position at 95% confidence level	2 positive (456, 478) and 111 negative sites	3 positive (52, 295, 425) and 59 negative sites	0 positive and 72 negative sites	1 positive (8) and 129 negative sites	2 positive (246, 574) and 114 negative sites	1 positive (614) and 439 negative sites
**Internal Branch Fixed Effect Likelihood (IFEL)**						
No. of positive and negative selection sites along with codon position at 95% confidence level	1 positive (456) and 61 negative sites	5 positive (52, 161, 285, 295, 425) and 44 negative sites	0 positive and 41 negative sites	1positive (8) and 68 negative sites	2 positive (246, 574) and 65 negative sites	2 positive (616, 623) and 218 negative sites
**Random Effects Likelihood (REL)**						
No. of positive and negative selection sites along with codon position at 10% confidence level	19 positive (46, 136, 160, 11, 375, 403, 423, 425, 426, 437, 435, 441, 447, 456, 467, 478, 484, 509, 517) and 209 negative sites	0 positive and 215 negative sites	0 positive and 02 negative sites	14 positive (5, 6, 8, 9, 11, 18, 46, 250, 299, 371, 411, 456, 518, 524) and 258 negative sites	0 positive and 339 negative sites	76 positive (35, 46, 81, 82, 93, 96, 120, 123, 124, 189, 194, 246, 279, 325, 334, 447, 455, 612, 613, 614, 617, 619, 620, 622, 623, 624, 627, 630, 631, 636, 641, 643, 645, 646, 647, 699, 720, 723, 798, 905, 928, 1004, 1031, 1116, 1185, 1257, 1264, 1280, 1375, 1390, 1401, 1547, 1551, 1649, 1655, 1660, 1698, 1700, 1710, 1722, 1725, 1747, 1783, 1840, 1918, 1976, 1980, 1995, 2010, 2029, 2135, 2142, 2144) and 160 negative sites
**Mixed Effect Model of Episodic Selection (MEME)**						
No. of selection sites and position of codon with evidences of episodic diversifying selection at 95% confidence level	4 sites (441, 456, 466, 478)	8 sites (10, 20, 83, 101, 102, 137, 403, 425)	3 sites (211, 311, 335)	5 sites (3, 8, 11, 46, 356)	7 sites (21, 210, 212, 288, 309, 330, 591)	17 sites (54, 68, 230, 349, 421, 455, 614, 719, 723, 1200, 1343, 1696, 1900, 1901, 2005, 2080, 2142)
**Fast Unbiased (FUBAR)**						
No. of false positive selection sites (Excluding to above mentioned sites) along with codon position at 95% confidence level	202 C.I (189–211)	293 C.I (264–312)	144 C.I (134-153)	208 C.I (200–222)	261 C.I (251–280)	779 C.I (765–802)

### Recombination analysis

**Figure 6:**
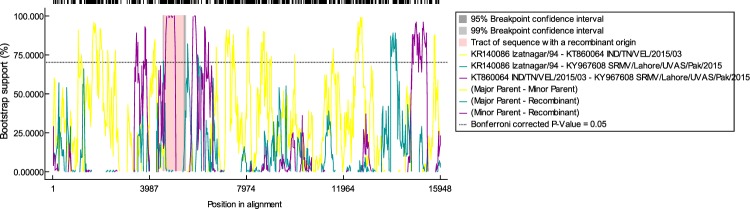
A graphical illustration of plot showing detection of recombination event.

Lying between 5ʹ UTR of the *M* gene (3406–4888 bp) and 3ʹ UTR of the *F* gene (4892–7306 bp), a putative recombination event was observed in the complete genome (4607–5425 nts) of Pakistan-origin strain of SRMV. With a probability of MC value of 2.357 E^−22^, this event was found between a recombinant Pakistani strain (KY967608; SRMV/Lahore/UVAS/Pak/2015) and Indian strains (KR140086; Izatngar/94 as major parent and KT860064; IND/TN/VEL/2015/03 as minor parent) (Fig. 6). This observation was consistent in all of the seven recombination algorithm methods at *p* < 0.001. A detailed information on inferred breakpoint and *p*-value of algorithm approaches is given in Table 10.

**Table 10:** Evidence of recombination events in the whole genome of Pakistan–originated SRMV strain along with breakpoint positions and significant *p*-values

Detecting Methods	*p*-value	Breakpoint position	SRMV Strains
RDP	4.360 × 10^−23^	Beginning breakpoint = 4607 nt Beginning breakpoint 90% C.I = 4556–4680 nt Ending breakpoint = 5425 nt Ending breakpoint 90% C.I = 5324–5504 nt Length of sequence between two breakpoint: 818 nt Binomial probability (MC corrected) = 2.357 E−22 Average ootstrap support = 89.83%	**Recombinant strain:** SRMV/Lahore/UVAS/Pak/2015 (KY967608) **Major Parent:** Izatnagar/94 (KR140086) (98.1% nucleotide identity) **Minor Parent:** IND/TN/VEL/2015/03 (KT860064) (99.8% nucleotide identity)
GENECONV	7.015 × 10^−21^		
BootScan	2.357 × 10^–22^		
MaxChi	2.278 × 10^−07^		
Chimaera	1.550 × 10^−06^		
SiScan	5.425 × 10^−13^		
3Seq	9.479 × 10^−11^		

## Discussion

We presented a comparative genetic, phylogenomic and evolutionary analysis of SRMV strains reported so far in public database. Whole genome sequences and open reading frames (ORFs) of individual genes of representative strains were used in subsequent higher-resolution bioinformatic analysis. This is because a specific gene might not evolve at the same rate as does the whole genome^15^ and, therefore, can provide precise information on viral evolutionary dynamics and necessary epidemiological investigations in future^16^. While considering the “rule of six” for whole gnome atlas, comparative complete genome analysis revealed a varying length of complete genome suggesting the potential of the virus to evolve over a period of time. A few sequences showed unsusual lengths (e.g., MF678816; 15927 bp, KM089831; 15957 bp and KM816619; 16058 bp) where, for each of these sequences, a nucleotide insertion/deletion was observed in the noncoding region between the *M* and *F* genes^17,18^. Interestetingly, each of these sequeunce was deriven from the next generation sequencing approach and, therefore, such an unusual length may correspond to the sequencing errors. Owing to the fact that all paramyxovirus including SRMV follow a polyhexameric genome length for the effective replication in host cells^19^, SRMV sequences erroneously not following the “rule of six” in genome atlas were excluded from the specific analysis.

Comparative residue analysis of viral proteins showed several conserved motifs^20,21^. Among these, the N protein had three conserved motifs. These included export signal, nuclear localization signal and RNA binding motif. The first two are considered responsible for transport of the N protein to nucleus of host cell, while the third one was believed to be involved in interaction of N-N monomers of RNA during genomic RNA binding and N-N self-interaction^20^. Developing polymerase complex with N and L proteins, the P protein plays a significant role in virus replication and RNA biosynthesis^22^. The protein contains a variable N-terminus whereas C-terminus is believed to be the most conserved, and is required for the interaction with L protein in synthesis of polymerase complex^23^. The Soyuz 1 motif and presence of ^151^S residue, responsible for viral transcription *via* altering its phosphorylation status^24^, were found in all study-included strains^21^. The M protein is a core organizer of viral morphogenesis and has the ability to interact with other proteins for maturation of viral progeny^25^. For all of the investigated strains, this protein carried a previously known residue pattern^21^ for late domain or cell membrane anchor, which has a known role for localization of cell membrane and budding activity^26^. An unusually long and GC rich non-coding region was observed between 3ʹ UTR-*M* and 5ʹ UTR-*F* genes in studied SRMV sequences. While no biological or functional significance is warranted, a previous study has suggested an up- and/or down-regulation of these proteins to differences in their lengths and therefore may alter cyto-pathogenicity and survival fitness of the virus in nature^27^.

Three motifs were also noticed in the F protein as signal peptide, cleavage site (responsible for virulence and adaptation in the environment) and a leucine zipper domain. These are known to be involved in maintenance of protein tertiary structure^20,22^. Since the signal peptide motif was located in a variable region^28^, we performed a comparative analysis to investigate the conserveness of specific residue at a specific position among all reported strains from different geographies and proposed a stretch of consensus residues at the global level. The H protein is considered responsible for attachment of the virus to host cell membrane *via* cleavage of sialic acid residue in cellular glycoprotein^29^. As observed in the current study, the protein has a hydrophobic domain at the N-terminus that acts as a signal peptide to anchor the protein into the membrane^20^. The findings of SLAM receptor binding sites during the analysis highlight the epitheliotropic and lymphotropic nature of SRMVs^30^. Herein, a high number of glycosylation sites were found in the N protein, which plays a major role in protein translocation^31^. The large protein (L) contributes in viral replication, transcription and polyadenylation using different domains that were found to be conserved in this study. Domain I, II and III are considered responsible for polymerase and kinase activity where GDDD and QGDNQ residues carry a prime significance^32^ and, as observed in a previous experimental study^33^, any substitution in these residues can abolish the polymerase activity of the L protein. Two highly conserved hinge regions were also observed in a pattern typically corresponding to established hinge regions of other closely related morbilliviruses^34^. Taken together, the potential influence of these substitutions in the functionality of corresponding proteins is scarce and, therefore, requires future investigations to determine impact of these variations in conserved domains. 

The phylogenetic analysis, either based upon complete genome or each of the complete coding genes, showed a clustering pattern according to distinct geographical setting and time-period e.g., strains clustered within a distinct clade represented same country of origin within a specific time period. Therefore, while presenting a global perspective, a clustering and subsequent sub-clade grouping is proposed in the current study as an imporved and updated version of previous proposal^35^. This is simply becuase the previous classification proposal was limited to sub-grouping of Indian strains along with a few of those reported from the Middle East and Africa. Not only that the said proposal excluded strains reported from China and Georgia but also did not represent a well-defined evolutionary cut-off for the lowest taxonomic node (sub-lineage or sub-grouping). In addition to that, Kumar *et al*.^35^ have classified the strains into clades and subclades which contradicts previously proposed standard classification criteria for the lowest taxonomic node or sub-grouping of the viruses within a lineage or genotype^36,37^. Though such a classification may provide some pre-liminary assessment exclusively for Indian-origin strains, a limited geographic-pattern based classification may raise controversies for SRMV classification at global scale. Therefore, these are considered unreliable to present molecular epidemiology of SRMVs worldwide. Indeed, with a substantial increase in the number of SRMV sequences in future, following a uniform classification criterion such as presented in the current study (IVa, IVb, IVc, IVd, IVe and IVf), is necessary for a more precise clustering at the lowest taxonomic node. While comparing different coding genes (*P*, *M*, *F*, *H* and *L*) of SRMV strains (Fig. 2a,b), minor differences were observed in the clustering pattern indicating an influence of nucleotides in genetic diversity of SRMVs. Nevertheless, the *N* gene-based topography was closer to those of the *L* gene (RNA-dependent RNA polymerase) and complete genome sequences. Thus it (*L *gene) could be employed alternatively for a precise evolutionary relationship of SRMV strains originating from different geographical regions. This is important because, considering SRMV a member of the family *Paramyxoviridae*, L protein is now considered as a standard criterion for classification of some of the closely related members of the sub-family *Avualvirinae*^38^. The observed topology of the *N* gene revealed evolutionary dynamics of circulating SRMV strains consistent with observations made previously^10,20^. Therefore, it is suggested that complete *N* and *L* gene-based phylogeny analysis can provide an accurate evolutionary relationship of the circulating strains in particular geographical settings^10,38^, especially for those regions where full-genomes have not yet been reported or have limited resources.

Nucleotide diversity analysis was used to unleash the genomic variation (polymorphism) within a given dataset^39^ where a substitution rate is considered a prime parameter to elucidate virus evolution over a period of time. The average number of pairwise nucleotide difference among the whole genome of all SRMV sequences was found to be 788.690 with a diversity in nucleotide sequences (0.04889 ± S.D. 0.00468) and haplotype variance (0.00001). Gained observations correspond to distinct features of RNA viruses where there is a lack of proofreading activity by reverse transcriptase^40^. In contrast to previous observations^14^, a lower diversity in nucleotide and haplotype variance, and nucleotide difference in the current study may largely be ascribed to inclusion of a smaller number of complete nucleotide sequences than those employed in the current study (n = 37 *vs* n = 68). In addition to this, evidenced by significant nucleotide diversity over a period of time (*p* < 0.05), the HKA test outcome indicated an ongoing evolution or adaptation of virus in the environment.

The DnaSP based nucleotide diversity analysis revealed higher diversity in the *H* gene than others of SRMVs. Owing to significant roles in attachment and subsequent genome replication, the gene has been proposed to assess the evolutionary relationship of SRMV strains^41^. Though it ascertains further research, the substitutions in the *H* gene may have an influence on host adaptability and pathogenicity to susceptible host such as observed previously for SRMV^14^ and influenza virus^42^. A diverse nucleotide hotspot was obsereved between 5ʹ UTR of *M* and 3ʹ UTR of the *F* genes in the whole genome. This aligns with observations made previously where a hotspot was identified at similar position between *M* and *F* genes^14^, highlighting potential variations in the genome size and corresponding substitutions^43^ in each of the gene. An influence of these spontaneous mutations in genome was assessed by employing Tajima’s D statistics that showed a non-significant negative value for all coding genes in DnaSP analysis, suggesting a lack of influence of spontaneous mutations on the fitness of individual virus. Such observations suggest positive selection among coding region of sequences with a lower level of sequence diversity and an excess of low-frequency variants reflecting the role of natural selection in SRMV genomes. Contrary to current study findings where analysis showed negative value for each of the coding genes, positive values in *F* and *H* genes has previously been suggested^14^.

The non-synonymous/synonymous rate (ω = dN-dS) is an important indicator of selective pressure at the protein level where ω = 1 means neutral mutations, ω < 1 correspond to purifying selection while ω > 1 indicates diversifying positive pressure^44^. Herein, as reported in a previous study^14^, the dN-dS plot for each protein showed value not more than 1 indicating a slow genetic evolution of SRMV. Indeed, such a comparison of rates of synonymous and non-synonymous mutations provides an understanding towards the mechanisms of molecular sequence evolution. The positive selection sites were found in all coding genes (*N*, *P*, *M*, *F*, *H* and *L*) using different statistical approaches. Though these sites were found to be non-significant with a ratio less than 1 by Tajima’s D statistics, it seldom happens in structural domains of genome. However, the impact of such positive selection sites with lower level of sequence diversity may cause the emergence of variants^44^. According to the neutral theory of molecular evolution, such type of molecular variations, which arise *via* spontaneous mutations, has no influence on individual’s fitness^45^. However, the biological significance of these sites still remains unknown and needs to be explored in future.

The occurrence of recombination events is considered a significant source of genetic diversity for RNA viruses^46^. Beside rare occurrence of recombination in negative sense RNA viruses particularly SRMV, an analysis for the detection of recombination event/s is recommended as a standard component of every phylogenetic analysis to serve an important quality-control function to weed out laboratory and analytical errors^47^. We found recombination events among Pakistani- and Indian-origin strains which further highlight the co-existence of similar SRMV strains along with its transboundary nature of transmission^48^. Indicating a high resolution of prediction, the observed putative recombination event was statistically significant and was identified by more than five recombination detection algorithms. Such an interference of Indian strains as major and minor parents for Pakistan-originated recombinant strain highlight its potential to cross international borders^48^. Similar finding has previously been observed for another RNA virus (*Yellow leaf* virus) from Pakistan and India^49^. Potnetial reason for such a sharing of genetic material could be spectulative and may be attributed to an increased disease incidence rate and frequent disease outbreaks near borderline of these countries^50,51^. Though potnetial occurrence of homologous recombination in some of the negative sense RNA viruses is low^52^, it is not surprising because sporadic recombination in various negative-sense RNA viuses such as Hantavirus^53,54^, ambisense arenaviruses^55,56^, Newcastle disease viruses^57,58^ and morbilliviruses (e.g. canine distemper virus^59^ and measles virus^60^) has been evidenced. Hence, an emergence of viral variants could be anticipated that may differ antigenically and serologically and therefore may have consequences in terms of failure in diagnotics and vaccine efficacy.

## Materials and Methods

### Complete genome sequencing of SRMVs from Pakistan and dataset information

The complete genome sequencing of two SRMV isolates [KY967609 (SRMV/Faisalabad/UVAS/Pak/2015) and KY967610 (SRMV/Layyah/UVAS/Pak/2015)] was performed as per primers and protocols described previously^5^. Later, including these two strains, a total of 75 whole genome sequences of SRMVs were accessed (https://www.ncbi.nlm.nih.gov/, October 01, 2019) and processed for subsequent bioinformatic analysis. Among these 75 SRMV sequences, four were attenuated vaccine strains (KJ867542, KF727981; HQ197753, X74443) and were excluded from the dataset used in the current study. Furtermore, given the “rule of six” genome atlas or polyhexameric genome length, 03 sequences including MF678816 (15927 bp), KM089831 (15957 bp) and KM816619 (16058 bp) were also excluded from comparative whole genome-specific analysis. However, owing to length of coding region comparable to each of the protein of SRMV, only the coding regions of these sequences were included and processed further in comparative genomic and residue analsyis. All essential information related to whole genome sequences of study-included strains is presented in Table 1.

### Comparative genomic analysis

The complete genome (15954 bp) dataset was aligned to equal length using ClustalW methods in BioEdit version 5.0.6^61^ and, based upon nucleotide number and position across the whole length of the genome, different genomic features were compared among all SRMV sequences. The consensus sequences were made for those SRMV sequences that had a highest nucleotide similarity and were originated from similar geographical regions. Nucleotide identity and divergence among all consensus whole genome sequences of lineages I-IV was assessed by Pairwise Sequence Comparisons (PASC) analysis in MEGA version 6.06^62^. The conserved domains, functional and structural motif/s, trans-membrane regions and unique substitutions in open reading frames were predicted using ORF Finder (http://www.ncbi.nlm.nih.gov/gorf/gorf.html), Conserved Domain Prediction tool (http://www.ncbi.nlm.nih.gov/Structure/cdd/wrpsb.cgi) and HMMTOP program (http://www.enzim.hu/hmmtop/index.php). The potential N-glycosylation sites (N-X-T/S, where X denoted any residue except a Proline) were predicted by NetNGlyc 1.0 server (http://www.cbs.dtu.dk/services/NetNGlyc) and accepted if the G-score was 0.5. Similarly, the diversity and/or conserveness of residues at important but hypervariable motif/s were analysed through WebLogo version 3.1 (accessible at http://weblogo.threeplusone.com/create.cgi).

### Estimation of evolutionary and divergence dates

Using a Bayesian Markov Chain Monte Carlo (MCMC) approach implemented in Bayesian evolutionary analysis sampling trees (BEAST) software package version 1.8.0^63^, the molecular evolutionary and divergence rates were co-estimated for complete genome and individual genes. For each dataset, a total of three independent runs of MCMC were conducted under a strict molecular clock model, using the Hasegawa–Kishino–Yano model of sequence evolution with a proportion of invariant sites and gamma distributed rate heterogeneity (HKY + I + C) with partitions into codon positions, and the remaining default parameters in the prior’s panel. For each gene, the MCMC run was 36107 steps long and the posterior probability distribution of the chains was sampled every 1000 steps. Convergence was assessed on the basis of an effective sampling size after 10% burn-in using Tracer software, version 1.5 (http://tree.bio.ed.ac.uk/software/tracer/). The estimations were the mean values obtained for the three runs. The mean time of the most recent common ancestor (TMRCA) and the 95% CI were calculated, and the best-fitting models were selected by a Bayes factor using marginal likelihoods implemented in Tracer^64^.

### Phylogeography-based reconstruction of evolutionary tree

A reliability of a gene for molecular epidemiology was assessed by comparing all coding genes (*N*, *P*, *M*, *F*, *H* and *L*) extracted from whole genome sequence of SRMV and aligned separately by ClustalW methods incorporated in the BioEdit version 5.0.6^61^. The phylogenetic trees were constructed by neighbour-joining method with best-fit substitution model for each set of sequences using MEGA version 6.06^62^. A 1000 replication bootstrap value was adjusted to better elucidate the probability and reliability of clustering of isolates or any change in their clustering pattern.

### Nucleotide diversity and natural selective pressure analysis

Based upon variable sites for mutations, and average numbers of pairwise nucleotide differences, the nucleotide diversity among coding sequences (CDS) of complete genome sequences was assessed for genomic polymorphism by DnaSP version 5.10.01 (accessible at http://www.ub.es/dnasp). The departure from neutrality in all isolate’s sequences was tested by Tajima’s D statistical method^65^. Divergence time in nucleotide diversity was estimated by a direct statistical model (HKA test). Data-monkey adaptive evolution server (http://www.datamonkey.org/) was used to evaluate synonymous (d_S_) and non-synonymous (d_N_) substitution rate per codon among CDS of all sequences^66^. Later, the positive and negative selection sites under natural selection were determined through six different genetic algorithms including Single Likelihood Ancestor Counting (SLAC), Fixed Effect Likelihood (FEL), Internal Branch Fixed Effect Likelihood (IFEL), Random Effects Likelihood (REL), Mixed Effect Model of Episodic selection (MEME) and Fast Unbiased Bayesien Approximation (FUBAR)^67^.

### Detection of putative recombination event

The sequences were analyzed for the identification of reliable putative breakpoints by different tools including SimPlot version 3.5.1^68^, GARD (http://www.datamonkey.org/GARD), DAMBE version 5.2.30^69^ and RDP4 version 4.95^70^. However, owing to an enhanced accuracy, clarity and reliability of analysis, outcomes gained by RDP4 were considered conclusive for further interpretation. The RDP4 was preferred because it employs a combination of seven different algorithms named RDP, GENECONV, BootScan, MaxChi, Chimaera, SiScan and 3Seq to better unleash putative recombinant and parent isolates at *p* < 0.001. A putative recombination event was assumed to have occurred only when it was consistently identified by at least four of the above-mentioned algorithms at a probability threshold of 0.05.

### Ethical approval and informed consent

This research did not involve human participants or animals. This article does not contain studies with animals or humans performed by any of the authors.
