# Author Correction: A comprehensive global perspective on phylogenomics and evolutionary dynamics of *Small ruminant morbillivirus*

**DOI:** 10.1038/s41598-020-78219-z

**Published:** 2020-11-27

**Authors:** Muhammad Zubair Shabbir, Aziz-ul Rahman, Muhammad Munir

**Affiliations:** 1 University of Veterinary and Animal Sciences, Lahore, 54600 Pakistan; 2 Division of Biomedical and Life Sciences, Lancaster University, Lancaster, LA1 4YG United Kingdom

Correction to: *Scientific Reports* 10.1038/s41598-019-54714-w, published online 08 January 2020

The original version of this Article contained errors.

After publication of the Article, it came to the Authors attention that when other sequence alignment tools are used, no recombination signal is identified. The Authors speculate that the positive identification of recombination events reported in the Article may have been caused by the highly variable non-coding region between M and F, which is hard to align. The Authors consider this identification to therefore be a bioinformatics error. Article was changed as follows to reflect this.

In the Results, ‘Recombination analysis’

“Lying between 5ʹ UTR of the *M* gene (3406–4888 bp) and 3ʹ UTR of the *F* gene (4892–7306 bp), a putative recombination event was observed in the complete genome (4607–5425 nts) of Pakistan-origin strain of SRMV. With a probability of MC value of 2.357 E^−22^, this event was found between a recombinant Pakistani strain (KY967608; SRMV/Lahore/UVAS/Pak/2015) and Indian strains (KR140086; Izatngar/94 as major parent and KT860064; IND/TN/VEL/2015/03 as minor parent) (Fig. 6). This observation was consistent in all of the seven recombination algorithm methods at *p* < 0.001. A detailed information on inferred breakpoint and *p*-value of algorithm approaches is given in Table 10.”

now reads:

“Lying between 5ʹ UTR of the *M* gene (3406–4888 bp) and 3ʹ UTR of the *F* gene (4892–7306 bp), apparently a putative recombination event was observed in the complete genome (4607–5425 nts) of Pakistan-origin strain of SRMV but it is attributed to bioinformatics errors. Therefore, no recombination was found in the current study.”

Additionally, Figure 6 and Table 10 were removed. The original figure and table are reproduced below.

**Figure 6:**
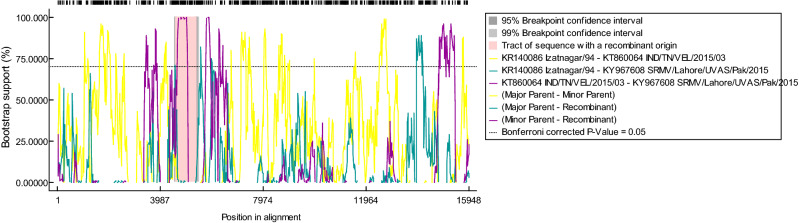
A graphical illustration of plot showing detection of recombination event.

**Table 10:** Evidence of recombination events in the whole genome of Pakistan-originated SRMV strain along with breakpoint positions and significant *p*-values.

Detecting Methods	*p*-value	Breakpoint position	SRMV Strains
RDP	4.360 × 10^−23^	Beginning breakpoint = 4607 nt Beginning breakpoint 90% C.I = 4556-4680 nt Ending breakpoint = 5425 nt Ending breakpoint 90% C.I = 5324-5504 nt Length of sequence between two breakpoint: 818 nt Binomial probability (MC corrected) = 2.357 E-22 Average ootstrap support = 89.83%	**Recombinant strain:** SRMV/Lahore/UVAS/Pak/2015 (KY967608) **Major Parent:** Izatnagar/94 (KR140086) (98.1% nucleotide identity) **Minor Parent:** IND/TN/VEL/2015/03 (KT860064) (99.8% nucleotide identity)
GENECONV	7.015 × 10^−21^		
BootScan	2.357 × 10^−22^		
MaxChi	2.278 × 10^−07^		
Chimaera	1.550 × 10^−06^		
SiScan	5.425 × 10^−13^		
3Seq	9.479 × 10^−11^		

In the Discussion,

“The occurrence of recombination events is considered a significant source of genetic diversity for RNA viruses^46^. Beside rare occurrence of recombination in negative sense RNA viruses particularly SRMV, an analysis for the detection of recombination event/s is recommended as a standard component of every phylogenetic analysis to serve an important quality-control function to weed out laboratory and analytical errors^47^. We found recombination events among Pakistani- and Indian-origin strains which further highlight the co-existence of similar SRMV strains along with its transboundary nature of transmission^48^. Indicating a high resolution of prediction, the observed putative recombination event was statistically significant and was identified by more than five recombination detection algorithms. Such an interference of Indian strains as major and minor parents for Pakistan-originated recombinant strain highlight its potential to cross international borders^48^. Similar finding has previously been observed for another RNA virus (*Yellow leaf* virus) from Pakistan and India^49^. Potnetial reason for such a sharing of genetic material could be speculative and may be attributed to an increased disease incidence rate and frequent disease outbreaks near borderline of these countries^50,51^. Though potnetial occurrence of homologous recombination in some of the negative sense RNA viruses is low^52^, it is not surprising because sporadic recombination in various negative-sense RNA viruses such as Hantavirus^53,54^, ambisense arenaviruses^55,56^, Newcastle disease viruses^57,58^ and morbilliviruses (e.g. canine distemper virus^59^ and measles virus^60^) has been evidenced. Hence, an emergence of viral variants could be anticipated that may differ antigenically and serologically and therefore may have consequences in terms of failure in diagnostics and vaccine efficacy.”

now reads:

“The occurrence of recombination events is considered a significant source of genetic diversity for RNA viruses^46^. Occurrence of recombination in negative-sense RNA viruses is extremely rare, still analysis for the detection of recombination event/s is recommended as a standard component of every phylogenetic analysis to serve an important quality-control function to weed out laboratory and analytical errors^47^. In the current study, no recombination event was found.”

As a result, the following References were removed and are listed below:

Seyoum, B. & Teshome, E. Major Transboundary Disease of Ruminants and their Economic Effect in Ethiopia. *Global J of Med Res* (2018).

Elsayed, A. I., Boulila, M., Odero, D. C. & Komor, E. Phylogenetic and recombination analysis of sorghum isolates of Sugarcane yellow leaf virus. *Plant Pathol* **67**(1), 221–32 (2018).

Muthuchelvan, D. *et al*. Molecular characterization of peste-des-petits ruminants virus (PPRV) isolated from an outbreak in the Indo-Bangladesh border of Tripura state of North-East India. *Vet Microbiol* **174**(3-4), 591–5 (2014).

Aziz-ul-R. *et al*. Evaluation of risk factors for peste des petits ruminants virus in sheep and goats at the Wildlife-Livestock Interface in Punjab Province, Pakistan. *BioMed Res Int* (2016).

Chare, E. R., Gould, E. A. & Holmes, E. C. Phylogenetic analysis reveals a low rate of homologous recombination in negative-sense RNA viruses. *J Gen Virol* **84**(10), 2691–703 (2003).

Klempa, B. *et al*. Genetic interaction between distinct Dobrava hantavirus subtypes in Apodemus agrarius and A. flavicollis in nature. *J Virol* **77**(1), 804–809 (2003).

Sironen, T., Vaheri, A. & Plyusnin, A. Molecular evolution of Puumala hantavirus. *J Virol* **75**(23), 11803–11810 (2001).

Charrel, R. N., de Lamballerie, X. & Fulhorst, C. F. The Whitewater Arroyo virus: natural evidence for genetic recombination among Tacaribe serocomplex viruses (family *Arenaviridae*). *Virology* **283**(2), 161–166 (2001).

Archer, A. M. & Rico-Hesse, R. High genetic divergence and recombination in Arenaviruses from the Americas. *Virology* **304**(2), 274–281 (2002).

Zhang, R., Wang, X., Su, J., Zhao, J. & Zhang, G. Isolation and analysis of two naturally-occurring multi-recombination Newcastle disease viruses in China. *Virus Res* **151**(1), 45–53 (2010).

Han, G. Z., He, C. Q., Ding, N. Z. & Ma, L. Y. Identification of a natural multi-recombinant of Newcastle disease virus. *Virology* **371**(1), 54–60 (2008).

Yuan, C. *et al*. Homologous recombination is a force in the evolution of canine distemper virus. *PloS one* **12**(4), e0175416 (2017).

Schierup, M. H., Mordhorst, C. H., Muller, C. P. & Christensen, L. S. Evidence of recombination among early-vaccination era measles virus strains. *BMC Evol Biol* **5**(1), 52 (2005).

Consequently, References 48–57 were incorrectly listed as References as 61–70.

These changes do not affect the overall conclusions of the Article. This has now been corrected in the PDF and HTML versions of the Article.

